# Understanding the Physiological Connection: Cardiac Arrest Following Prone Positioning

**DOI:** 10.7759/cureus.63050

**Published:** 2024-06-24

**Authors:** Taizoon Q Dhoon, Anil Tiwari, Evan Villaluz, Debra E Morrison

**Affiliations:** 1 Anesthesiology, University of California, Irvine (UCI) Health, Orange, USA; 2 Anesthesiology and Perioperative Medicine, University of California, Irvine (UCI) Health, Orange, USA

**Keywords:** patient position, diagnostic and therapeutic ercp, quality improvement and patient safety, patient safety, prone positioning

## Abstract

Prone positioning of patients is a routine occurrence in procedural suites and operating rooms (ORs). However, the physiological changes that occur with prone positioning are frequently underappreciated by proceduralists, surgeons, and anesthesiologists. This may be related to a sense of the routine or a lack of familiarity with physiological changes that accompany the prone position.

The prone position, while aiding visualization and cannulation of the ampulla of Vater during endoscopic retrograde cholangiopancreatography (ERCP), can induce physiological changes such as reduced preload, inferior vena cava filling, and cardiac output; it can also increase intrathoracic pressure and mediastinal compression. Anesthetic agents can further impact cardiopulmonary physiology, decreasing systemic vascular resistance and reducing cardiac contractility. In addition, the transition from negative to positive pressure ventilation following endotracheal intubation can increase pulmonary artery pressures and right ventricular (RV) strain. Therefore, caution is needed with patients who have RV dysfunction, pulmonary hypertension, or preload dependency, as they may not tolerate prone positioning.

We describe a case in which a 73-year-old male patient scheduled for an ERCP suffered cardiac arrest after being transitioned to the prone position. The patient was repositioned in the supine position and resuscitated. The case was completed in the supine position.

## Introduction

Prone positioning of patients is a routine occurrence in procedural suites and operating rooms (ORs). However, the physiological changes that occur with prone positioning are frequently underappreciated by proceduralists, surgeons, and anesthesiologists. This may be related to a sense of the routine or a lack of familiarity with physiological changes that accompany the prone position.

Endoscopic retrograde cholangiopancreatography (ERCP), for example, which became more established in the late 1960s, has historically favored the prone position due to improved visualization and cannulation of the ampulla of Vater. Although the procedure can be performed successfully in the left lateral or supine position, the prone position is desirable [1,2]. However, the prone position induces various physiological changes, particularly in cardiovascular function, that, if ignored, can lead to potential intraoperative complications.

Physiological alterations associated with prone positioning include reduced preload, inferior vena cava filling, and cardiac output. The prone position can also increase intrathoracic pressure and mediastinal compression [1-3]. Anesthetic agents can further impact cardiopulmonary physiology, decreasing systemic vascular resistance and reducing cardiac contractility [4,5]. Additionally, the transition from negative to positive pressure ventilation following endotracheal intubation can increase pulmonary artery pressures and right ventricular (RV) strain. Therefore, those with RV dysfunction, pulmonary hypertension, or preload dependency may not tolerate prone positioning [6].

Additional physiological changes include elevated heart rate, increased afterload, and reduced preload and cardiac contractility [3]. These effects can be seen with direct mechanical compression of the thorax or the abdomen. Direct inward force of the sternum leads to compression of the mediastinum in the prone position and can lead to RV compromise [3]. The prone position can also increase intrathoracic pressure, thereby reducing inferior vena cava filling, atrial filling, and LV compliance with a subsequent decrease in cardiac output [4,5]. Prone positioning in healthy adult males demonstrated a 7.3% increase in heart rate, while the transaortic flow velocity decreased by 13.7% [7].

In addition to chest wall and sternal compression, compression of the abdomen in the prone position can profoundly reduce RV function as well as catastrophic reduction inferior vena cava preload. Abdominal compression with hemodynamic compromise is most notable in patients with obesity [4]. This can result in decreased arterial inflow and venous outflow to the visceral organs, reduced preload and central blood volume, and sequestration of blood in dependent body parts [4].

The prone position may place patients in unforeseen code blue scenarios when patients with specific risk factors are not properly identified. Risk factors for cardiovascular collapse in the prone position include patients with a history of restrictive or obstructive lung disease, scoliosis, pectus excavatum, or recent cardiothoracic surgery [3,6]. The prone position can be a precarious hemodynamic scenario, and these patients may be at heightened risk of myocardial infarction and cardiac arrest during and after positioning [3,6].

In addition, anesthetic choice can have a profound effect on cardiopulmonary physiology. Anesthetic agents (e.g., volatile agent and propofol) are known to decrease systemic vascular resistance, alter heart rate, and decrease cardiac contractility. Etomidate may be considered due to its reduced risk of myocardial depression but has been associated with adrenal suppression and a potential increase in mortality in critically ill patients, particularly in patients with sepsis [8].

In cases requiring general anesthesia (note that general endotracheal anesthesia (GETA) is often indicated when the patient is in the prone position, since a delay in rescuing the airway mid-procedure may lead to hypoxia or anoxia), the transition from negative pressure ventilation to positive pressure ventilation, with or without positive end-expiratory pressure (PEEP), can lead to increased pulmonary artery pressures and RV strain. Extreme care should be taken with patients with RV dysfunction and pulmonary hypertension or in patients who are preload-dependent, as these patients may not tolerate the RV strain associated with prolonged prone positioning [6].

## Case presentation

A 73-year-old male, American Society of Anesthesiologists (ASA) physical status class IV, with a history of severe aortic stenosis and chronic atrial fibrillation (Afib) had undergone a successful transcatheter aortic valve replacement (TAVR). Post-procedural transthoracic echo (TTE) noted normal left ventricular (LV) systolic function, with an LV ejection fraction (LVEF) of 59.5%; moderate concentric LV hypertrophy; left atrium (LA) moderately dilated; normal right ventricular (RV) size and systolic function; and mildly dilated right atrium (RA). The 26-mm Edwards SAPIEN 3 Ultra RESILIA (Edwards Lifesciences, Irvine, CA) was in the aortic valve position, with normal transvalvular gradients and without a paravalvular leak.

Incidentally, during the TAVR workup, computed tomography (CT) imaging noted that the patient had a pancreatic mass. Following a multidisciplinary discussion, the decision was made to proceed with the TAVR due to the severity of aortic stenosis and the fact that the patient was asymptomatic from the mass at that time. While recovering at home, the patient began to experience worsening abdominal pain (19 days after TAVR). He was admitted to the hospital and diagnosed with sepsis due to cholangitis.

The patient underwent an initial ERCP two days later. He tolerated general anesthesia in the prone position with vasopressor support (phenylephrine 400 mcg and vasopressin one unit IV over a 90-minute period). Following the initial ERCP and stent placement, the patient continued to be treated for sepsis and post-ERCP pancreatitis. Following the procedure, the patient's aspartate aminotransferase (AST), alanine aminotransferase (ALT), and alkaline phosphatase (ALP) levels remained stable. Despite the intervention, the patient continued to experience moderate abdominal pain.

Four days later, the patient developed progressive encephalopathy, Afib with rapid ventricular response (RVR) in the 150s, and hypotension. The patient was admitted to the intensive care unit (ICU) where an IV fluid bolus and vasopressors were initiated (norepinephrine 20 mcg/minute). The patient was transported to the operating room (OR) for an ERCP to evaluate the cholangitis, with hopes of improving the sepsis with a stent.

Upon arrival at the OR, the patient was placed on standard ASA monitors. Gastroenterology requested prone positioning with general endotracheal anesthesia. Anesthesia was induced with propofol 0.5 mg/kg and rocuronium 1 mg/kg, followed by uneventful intubation. The patient was hemodynamically stable following induction. The patient received sevoflurane for maintenance of anesthesia, and the norepinephrine infusion was maintained at 20 mcg/minute throughout the case. The team discussed the placement of a pre-induction arterial catheter. However, the plan was to insert it while the patient was in a prone position, with the expectation that prompt intervention from the ERCP may improve the patient's septic shock.

Minutes after intubation, the patient was flipped into the prone position. Weak pulses were noted following prone positioning, and resuscitation began with additional IV fluids, norepinephrine, and epinephrine. Hypotension ensued and was refractory to vasopressor boluses. Given the hemodynamic instability, the patient was returned to the supine position, chest compressions commenced, and return of spontaneous circulation (ROSC) was obtained. An arterial and central venous catheter were placed, and the patient was stabilized. Following a discussion between the anesthesiologist and gastroenterologist, the case was successfully completed in the supine position. A covered metal stent was inserted into the common bile duct (CBD) to mitigate a 4 cm distal CBD stenosis. Following the stent placement, a stenotic area was observed within the stent. This was addressed by performing an 8 mm balloon dilation of the stenosis through the stent.

**Figure 1:**
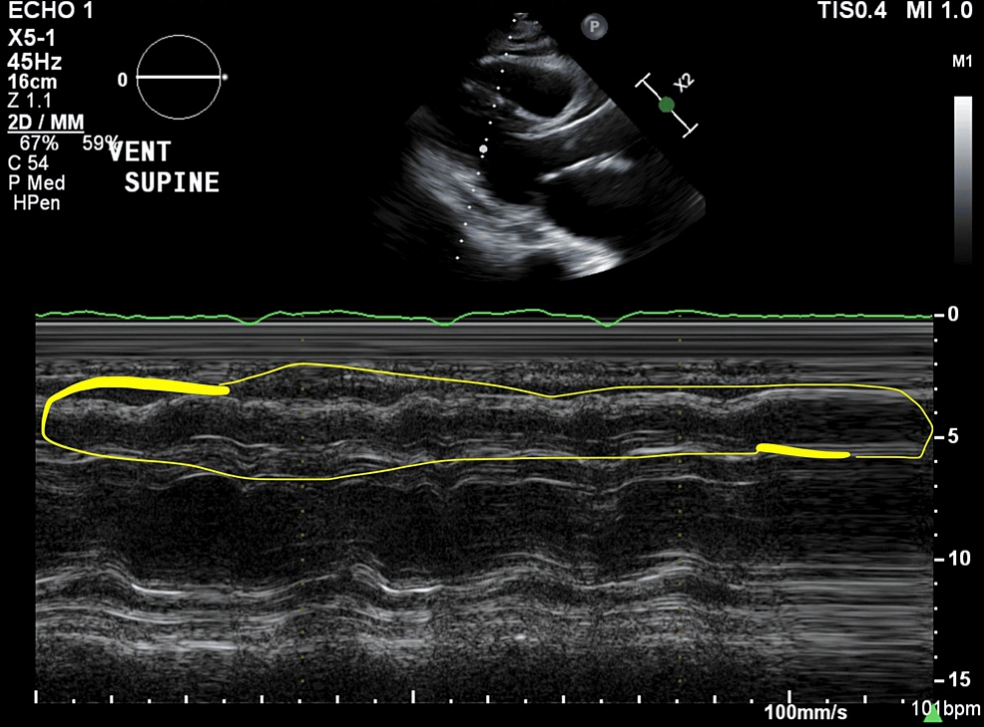
TTE image (parasternal long-axis view) The area circled shows a time-lapse image of the RV walls contracting and mild-moderate RV dysfunction. TTE: transthoracic echo, RV: right ventricular

The patient was then transferred to the ICU, intubated, sedated, and remained on a norepinephrine infusion of 20 mcg/minute. Following ICU admission, a TTE showed no wall motion abnormalities and an LVEF of 52%, as well as new moderate LV diastolic dysfunction, moderate RV systolic dysfunction, and moderately elevated pulmonary artery systolic pressures (Figure 1). These echocardiographic changes from post-TAVR TTE results were attributed to severe septic shock. The patient expired five days later due to complications of severe septic shock.

This case report was exempted from institutional review board (IRB) oversight.

## Discussion

Preoperative recommendations

A thorough and focused preoperative examination is warranted for all patients scheduled in the prone position. The preoperative discussion should include the projected duration of the procedure and proposed patient positioning. The patient's capacity to handle the prone position should be carefully evaluated. If cardiac testing is warranted, a workup not only provides a risk assessment for ischemic heart disease but also provides an assessment of RV function, pulmonary hypertension, and valvular disease [5].

The discussion concerning patient positioning, especially for high-risk patients, should be a collaborative effort involving the proceduralist, surgeon, and anesthesiologist. The prone position also increases the likelihood of mediastinal compression, as the right ventricle can be compressed against the sternum [1]. The prone position can also increase intrathoracic pressure, reduce inferior vena cava filling, reduce atrial filling, and reduce left ventricular compliance with subsequent decrease in cardiac output. The use of bolsters (specially designed support devices or padding used to maintain a patient's position during surgery) in the setting of chest and abdominal positioning is crucial. Local compression of the anterior chest wall or abdomen may catastrophically reduce right ventricular function or inferior vena cava preload, respectively. Patients with scoliosis, pectus excavatum, or recent cardiothoracic surgery may be at heightened risk [1]. In addition, anesthetic agents can have a profound effect on cardiopulmonary physiology. Volatile anesthetics and propofol are known to decrease systemic vascular resistance, alter heart rate, and decrease cardiac contractility (due to direct myocardial suppression).

Risk factors for a cardiovascular collapse in the prone position include massive blood loss, hypothermia, fluid shifts, cardiac comorbidities, venous air embolism, and anatomic deformities (e.g., thoracic lordosis or pectus excavatum) [1-3]. Extreme care should be taken with patients with right ventricular dysfunction and pulmonary hypertension or in patients who are preload-dependent or highly sensitive to elevations in pulmonary vascular resistance [6]. These patients may not tolerate the right ventricular strain associated with prolonged prone positioning [5]. A thorough discussion should be undertaken to determine the safety of the prone position. This patient subset may be at a heightened risk of myocardial infarction and cardiac arrest when transitioned to the prone position.

In patients who may not tolerate the prone position, it may be judicious to attempt the desired position out of the OR in advance of the day of surgery, setting up the operating table or gurney with the patient in comfortable clothes and attempting to position the patient in the desired position. If prone positioning is not suitable, consider whether the lateral or supine position would be more appropriate.

Management recommendations

In cases where the prone position is unavoidable, a meticulously crafted management plan should be in place, developed jointly by the proceduralist and anesthesiologist. In high-risk patients, the need for a central venous catheter, arterial catheter, inotropes, or vasopressors should be determined. All vascular access should be obtained prior to prone positioning. If hemodynamic lability is anticipated following the position change, inotropes or vasopressors may need to be initiated prior to the transition to the prone position.

The choice between monitored anesthesia care (MAC) and general endotracheal anesthesia (GETA) is a critical decision in the treatment process. MAC is typically preferred for shorter, routine procedures, while GETA is often used for lengthier, more complex operations. The choice of GETA is most notable in patients with a heightened risk of pulmonary aspiration [9]. Concerningly, the risks of hypotension and reduced cardiac output are higher with GETA compared to MAC, making the prone position an added layer of consideration. This is evidenced by a comprehensive observational study, which demonstrated that hypotension occurred in 59% of patients who underwent general anesthesia for ERCP, compared with only 14% of those who received monitored anesthesia care [9-12].

Implementation of chest and abdomen bolsters is crucial when positioning to avoid compression of the chest and abdomen. Careful placement of the bolsters reduced the risk of RV compression, elevations in intrathoracic pressure, and reduction in preload. Following positioning in the prone position, two blood pressure readings should be obtained over a short interval, and the vital signs closely monitored. The patient's original bed/gurney should be kept in the room until hemodynamic stability has been noted following prone positioning. Once hemodynamic stability is noted by following positioning, the bed/gurney can be removed. In the event the patient does not tolerate the change of position, immediate availability of the bed facilitates quick supination of the patient and prompt resuscitation.

Case review 

In our case, the cardiac complications that arose after the transition to the prone position were likely due to multiple factors. The physiological disturbances that occurred post-induction of anesthesia may have led to direct myocardial depression and a decrease in systemic vascular resistance, although the patient's initial blood pressure readings were stable in the supine position. The subsequent switch to positive pressure ventilation, following endotracheal intubation, may have led to an increase in RV workload and pulmonary arterial pressures, as well as decreased preload due to elevations in intrathoracic pressure.

In the context of severe septic shock, these physiological disturbances can be more pronounced and potentially harmful. Sepsis can lead to increases in pulmonary artery pressure and can cause RV dysfunction. When these factors were combined with the physiological changes following induction of anesthesia and positive pressure ventilation, the risk of complications was significantly increased.

When the patient was transitioned to the prone position, it is probable that he experienced chest wall compression, leading to reduced right ventricular (RV) function. Additionally, abdominal compression may have potentiated a reduction in the preload via the inferior vena cava. These combined physiological changes could have led to hemodynamic instability and cardiac arrest following prone positioning. Therefore, transitioning patients, particularly those in critical conditions, to the prone position should be done with utmost care to reduce the likelihood of severe complications.

The physiological disturbances that ensued post-induction of anesthesia, endotracheal intubation, and the subsequent switch to positive pressure ventilation, along with reductions in preload, RV compression, and elevations in intrathoracic pressure in the prone position, all contributed. These factors, combined with the patient's severe septic shock, leading to increases in pulmonary artery pressure and moderate RV dysfunction, significantly increased the risk of complications (Figure 1). Upon transition to the prone position, the patient likely experienced chest wall compression and abdominal compression, reducing RV function and inferior vena cava preload, respectively. A combination of these physiological changes likely resulted in hemodynamic instability and cardiac arrest when transitioned to the prone position.

## Conclusions

The complications that can arise from the prone position are often underestimated. Proceduralists, surgeons, and anesthesiologists need to be thoroughly knowledgeable about the common physiological changes that can occur when the prone position is utilized. It is crucial to have a comprehensive discussion about high-risk patients, assess their capacity to tolerate the prone position, and create a plan that minimizes potential risk to the patient. Physicians should consider alternative positions, such as the lateral or supine positions, when possible. When the prone position is necessary for the aforementioned high-risk patients, it is recommended to secure arterial and venous access while the patient is in the supine position. Inotropes and vasopressors should be readily available in the room and may need to be initiated before transitioning to the prone position to prevent hemodynamic instability. Once a patient is positioned prone, it is essential to promptly and judiciously monitor their vital signs, keeping the original bed or gurney within the room until hemodynamic stability is established and the patient is seen to tolerate the prone position. In the event of intolerance to the positional change, the patient can be swiftly reverted to the supine position for resuscitation. Once hemodynamic stability post-positional change is confirmed, the bed or gurney can be removed.

We delineate how thoughtful planning and dialogue with the entire care team may help prevent anticipated complications and provide an additional level of safety for the patient.
